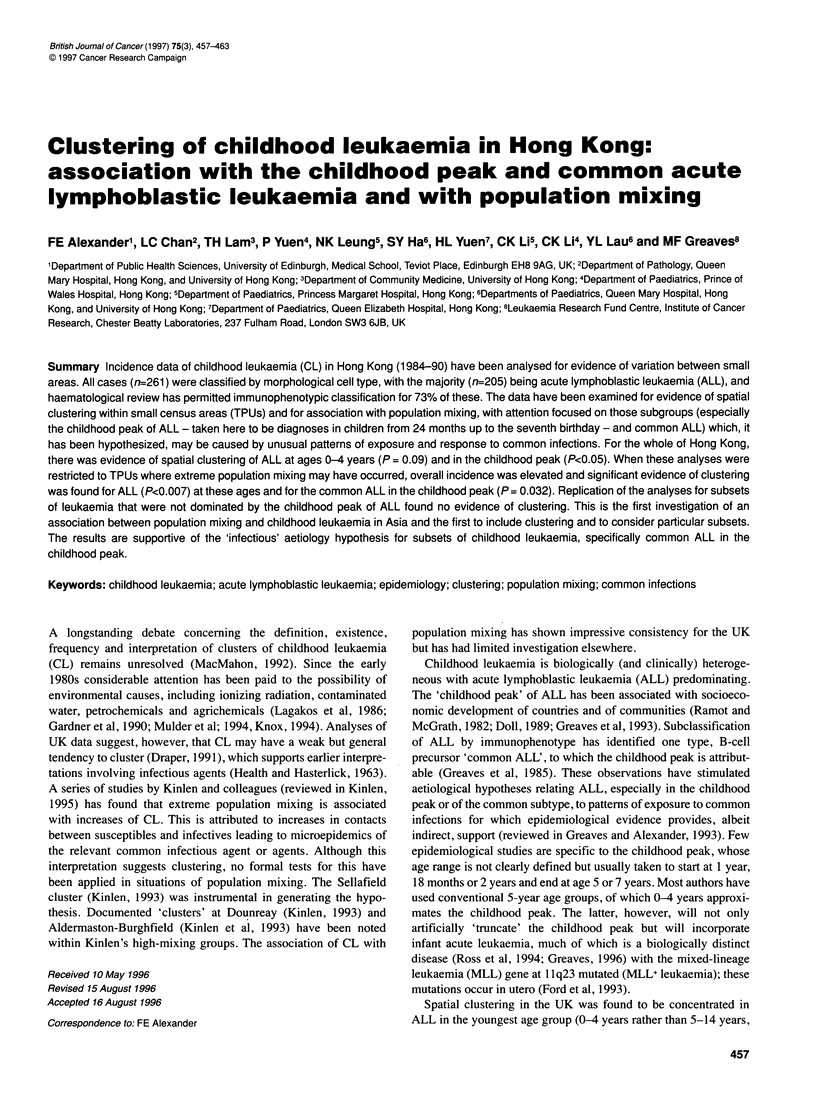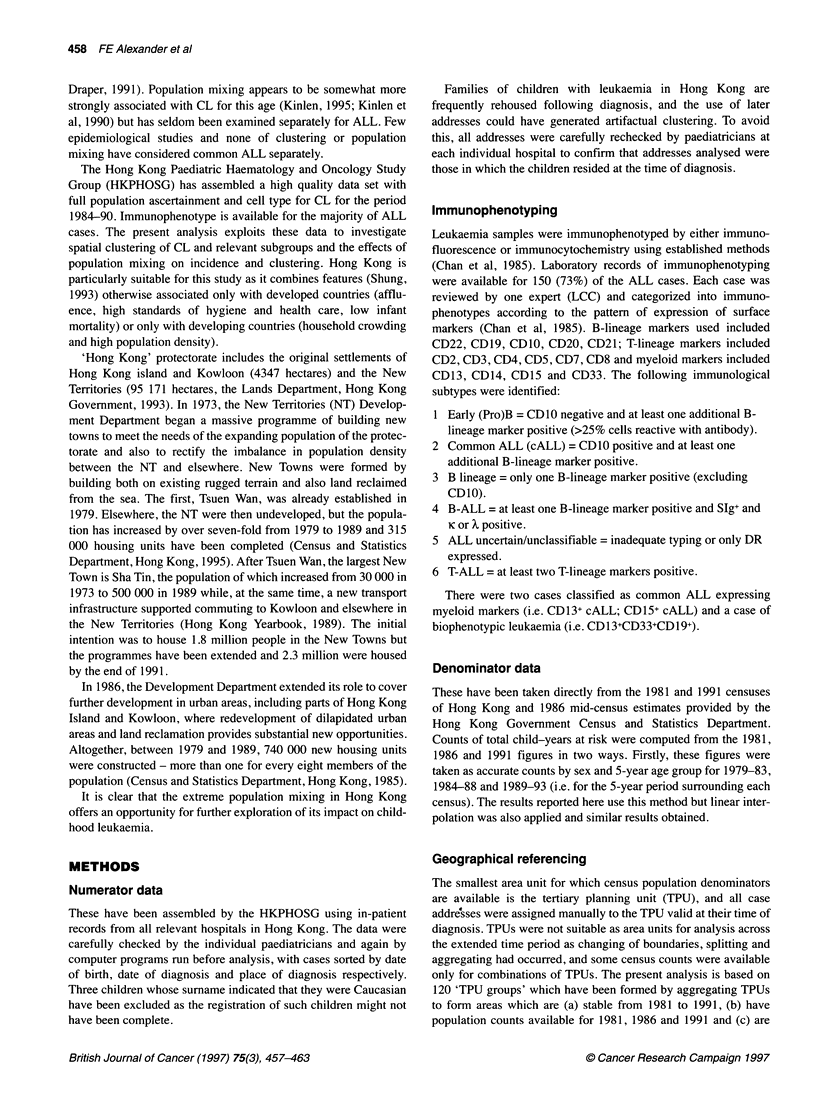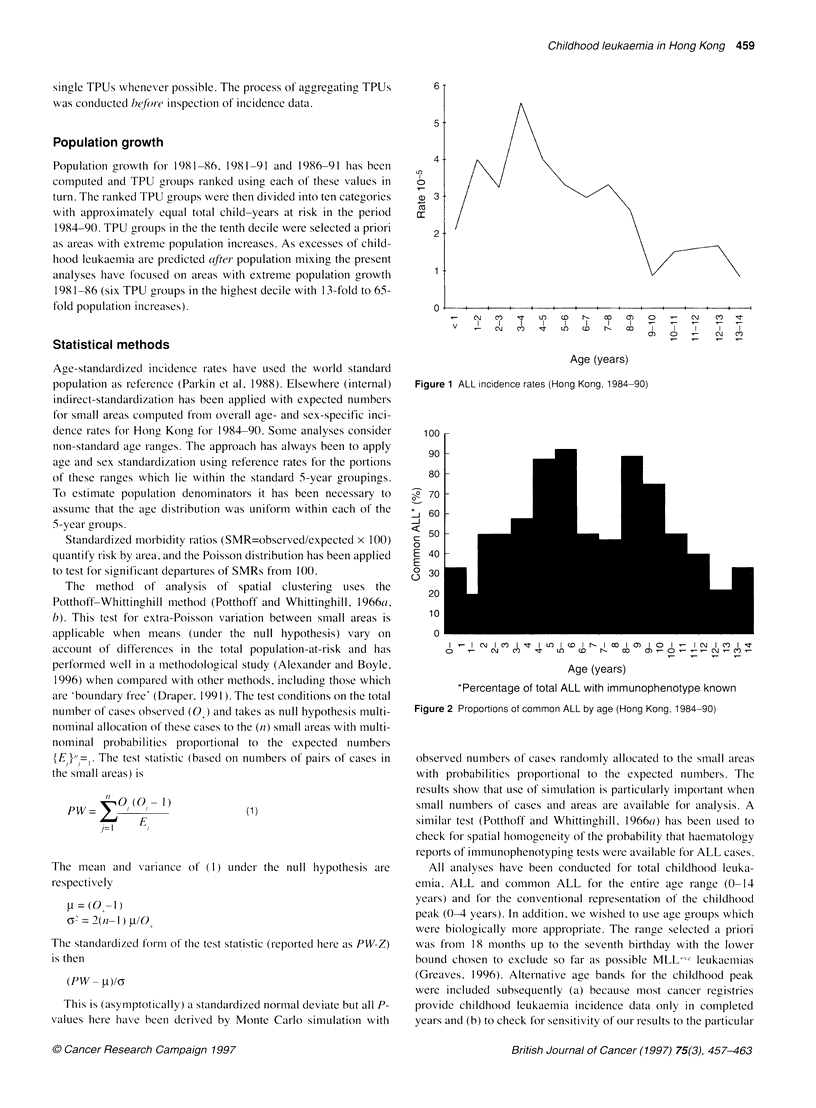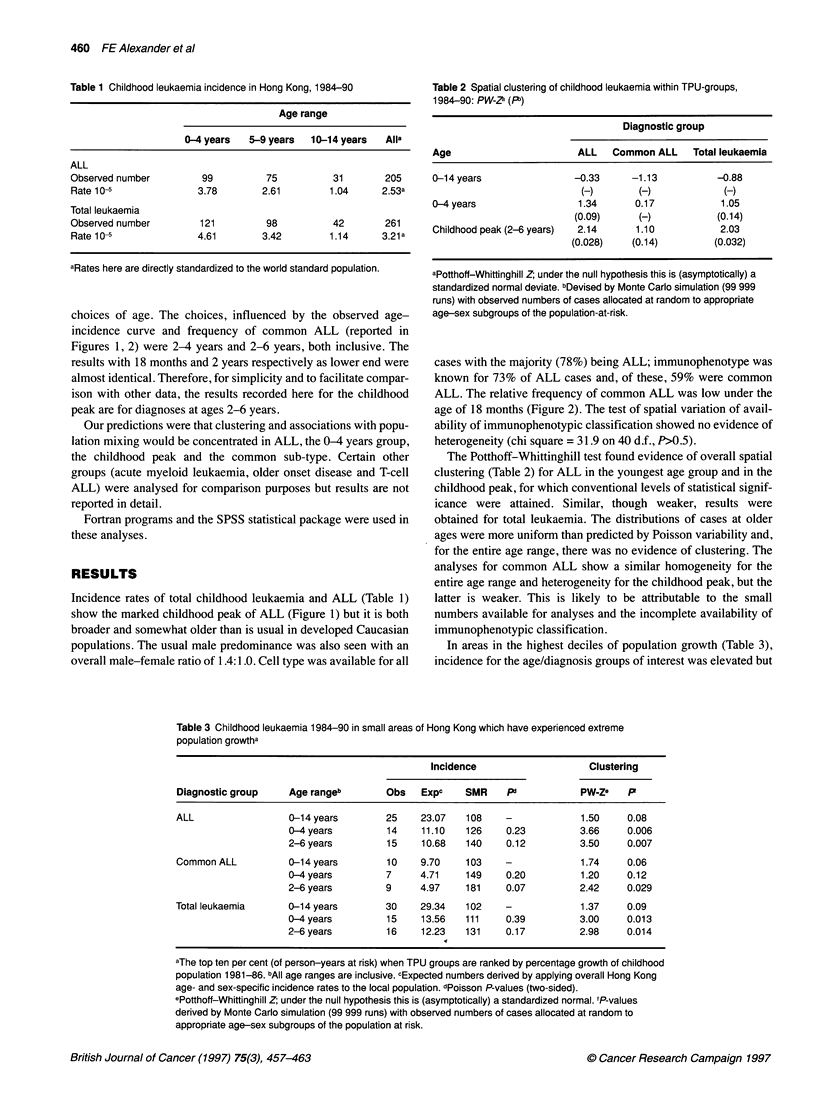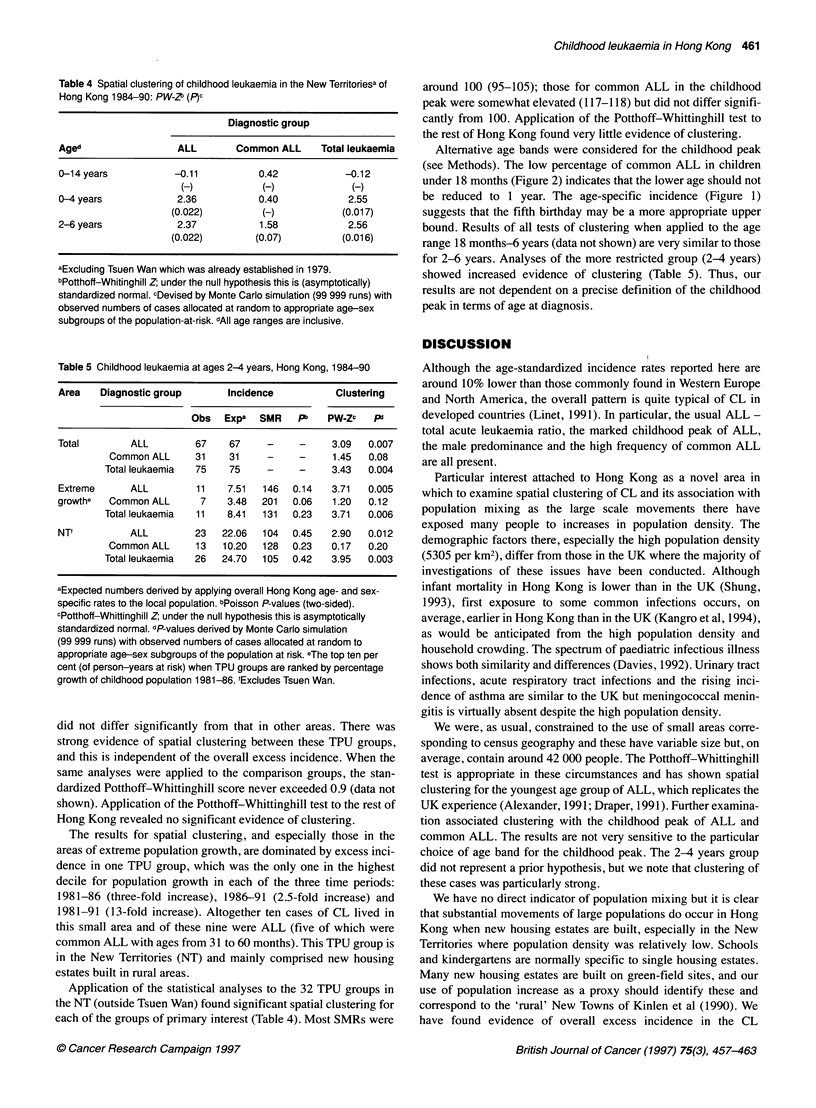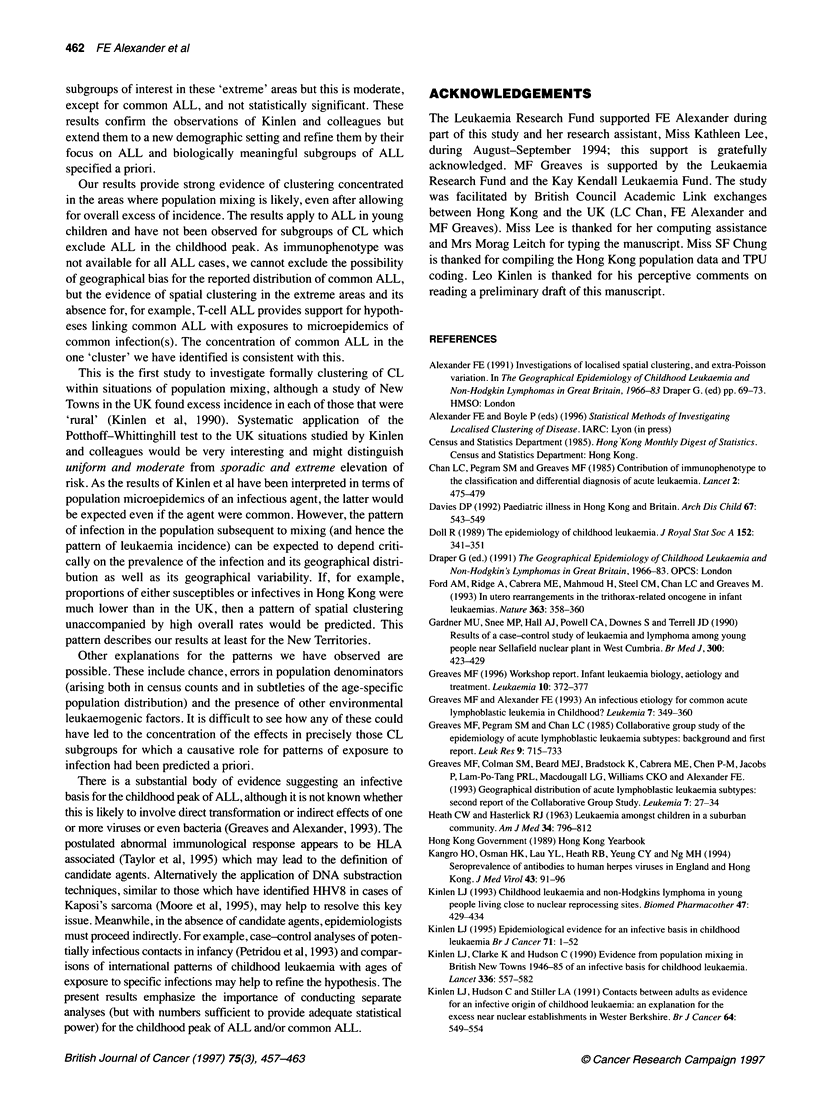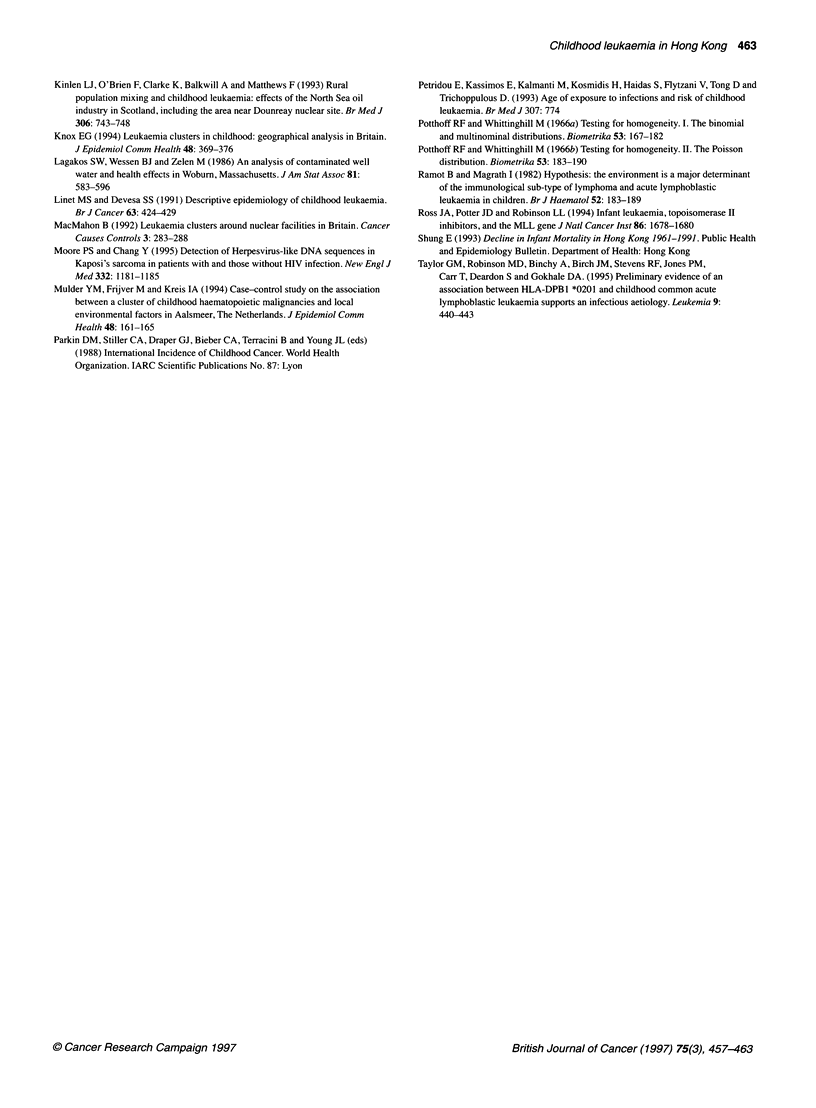# Clustering of childhood leukaemia in Hong Kong: association with the childhood peak and common acute lymphoblastic leukaemia and with population mixing.

**DOI:** 10.1038/bjc.1997.77

**Published:** 1997

**Authors:** F. E. Alexander, L. C. Chan, T. H. Lam, P. Yuen, N. K. Leung, S. Y. Ha, H. L. Yuen, C. K. Li, C. K. Li, Y. L. Lau, M. F. Greaves

**Affiliations:** Department of Public Health Sciences, University of Edinburgh, Medical School, Teviot Place, UK.

## Abstract

Incidence data of childhood leukaemia (CL) in Hong Kong (1984-90) have been analysed for evidence of variation between small areas. All cases (n=261) were classified by morphological cell type, with the majority (n=205) being acute lymphoblastic leukaemia (ALL), and haematological review has permitted immunophenotypic classification for 73% of these. The data have been examined for evidence of spatial clustering within small census areas (TPUs) and for association with population mixing, with attention focused on those subgroups (especially the childhood peak of ALL--taken here to be diagnoses in children from 24 months up to the seventh birthday--and common ALL) which, it has been hypothesized, may be caused by unusual patterns of exposure and response to common infections. For the whole of Hong Kong, there was evidence of spatial clustering of ALL at ages 0-4 years (P = 0.09) and in the childhood peak (P<0.05). When these analyses were restricted to TPUs where extreme population mixing may have occurred, overall incidence was elevated and significant evidence of clustering was found for ALL (P<0.007) at these ages and for the common ALL in the childhood peak (P = 0.032). Replication of the analyses for subsets of leukaemia that were not dominated by the childhood peak of ALL found no evidence of clustering. This is the first investigation of an association between population mixing and childhood leukaemia in Asia and the first to include clustering and to consider particular subsets. The results are supportive of the 'infectious' aetiology hypothesis for subsets of childhood leukaemia, specifically common ALL in the childhood peak.


British Journal of Cancer (1997) 75(3), 457–463
© 1997 Cancer Research Campaign

### Clustering of childhood leukaemia in Hong Kong: association with the childhood peak and common acute lymphoblastic leukaemia and with population mixing

FE Alexander[1], LC Chan[2], TH Lam[3], P Yuen[4], NK Leung[5], SY Ha[6], HL Yuen[7], CK Li[5], CK Li[4], YL Lau[6] and MF Greaves[8]

[1]Department of Public Health Sciences, University of Edinburgh, Medical School, Teviot Place, Edinburgh EH8 9AG, UK; [2]Department of Pathology, Queen Mary Hospital, Hong Kong, and University of Hong Kong; [3]Department of Community Medicine, University of Hong Kong; [4]Department of Paediatrics, Prince of Wales Hospital, Hong Kong; [5]Department of Paediatrics, Princess Margaret Hospital, Hong Kong; [6]Departments of Paediatrics, Queen Mary Hospital, Hong Kong, and University of Hong Kong; [7]Department of Paediatrics, Queen Elizabeth Hospital, Hong Kong; [8]Leukaemia Research Fund Centre, Institute of Cancer Research, Chester Beatty Laboratories, 237 Fulham Road, London SW3 6JB, UK

**Summary** Incidence data of childhood leukaemia (CL) in Hong Kong (1984–90) have been analysed for evidence of variation between small areas. All cases ($n$=261) were classified by morphological cell type, with the majority ($n$=205) being acute lymphoblastic leukaemia (ALL), and haematological review has permitted immunophenotypic classification for 73% of these. The data have been examined for evidence of spatial clustering within small census areas (TPUs) and for association with population mixing, with attention focused on those subgroups (especially the childhood peak of ALL – taken here to be diagnoses in children from 24 months up to the seventh birthday – and common ALL) which, it has been hypothesized, may be caused by unusual patterns of exposure and response to common infections. For the whole of Hong Kong, there was evidence of spatial clustering of ALL at ages 0–4 years ($P$ = 0.09) and in the childhood peak ($P$<0.05). When these analyses were restricted to TPUs where extreme population mixing may have occurred, overall incidence was elevated and significant evidence of clustering was found for ALL ($P$<0.007) at these ages and for the common ALL in the childhood peak ($P$ = 0.032). Replication of the analyses for subsets of leukaemia that were not dominated by the childhood peak of ALL found no evidence of clustering. This is the first investigation of an association between population mixing and childhood leukaemia in Asia and the first to include clustering and to consider particular subsets. The results are supportive of the 'infectious' aetiology hypothesis for subsets of childhood leukaemia, specifically common ALL in the childhood peak.

**Keywords:** childhood leukaemia; acute lymphoblastic leukaemia; epidemiology; clustering; population mixing; common infections

A longstanding debate concerning the definition, existence, frequency and interpretation of clusters of childhood leukaemia (CL) remains unresolved (MacMahon, 1992). Since the early 1980s considerable attention has been paid to the possibility of environmental causes, including ionizing radiation, contaminated water, petrochemicals and agrichemicals (Lagakos et al, 1986; Gardner et al, 1990; Mulder et al; 1994, Knox, 1994). Analyses of UK data suggest, however, that CL may have a weak but general tendency to cluster (Draper, 1991), which supports earlier interpretations involving infectious agents (Health and Hasterlick, 1963). A series of studies by Kinlen and colleagues (reviewed in Kinlen, 1995) has found that extreme population mixing is associated with increases of CL. This is attributed to increases in contacts between susceptibles and infectives leading to microepidemics of the relevant common infectious agent or agents. Although this interpretation suggests clustering, no formal tests for this have been applied in situations of population mixing. The Sellafield cluster (Kinlen, 1993) was instrumental in generating the hypothesis. Documented 'clusters' at Dounreay (Kinlen, 1993) and Aldermaston-Burghfield (Kinlen et al, 1993) have been noted within Kinlen's high-mixing groups. The association of CL with population mixing has shown impressive consistency for the UK but has had limited investigation elsewhere.

*Received 10 May 1996*
*Revised 15 August 1996*
*Accepted 16 August 1996*

*Correspondence to:* FE Alexander

Childhood leukaemia is biologically (and clinically) heterogeneous with acute lymphoblastic leukaemia (ALL) predominating. The 'childhood peak' of ALL has been associated with socioeconomic development of countries and of communities (Ramot and McGrath, 1982; Doll, 1989; Greaves et al, 1993). Subclassification of ALL by immunophenotype has identified one type, B-cell precursor 'common ALL', to which the childhood peak is attributable (Greaves et al, 1985). These observations have stimulated aetiological hypotheses relating ALL, especially in the childhood peak or of the common subtype, to patterns of exposure to common infections for which epidemiological evidence provides, albeit indirect, support (reviewed in Greaves and Alexander, 1993). Few epidemiological studies are specific to the childhood peak, whose age range is not clearly defined but usually taken to start at 1 year, 18 months or 2 years and end at age 5 or 7 years. Most authors have used conventional 5-year age groups, of which 0–4 years approximates the childhood peak. The latter, however, will not only artificially 'truncate' the childhood peak but will incorporate infant acute leukaemia, much of which is a biologically distinct disease (Ross et al, 1994; Greaves, 1996) with the mixed-lineage leukaemia (MLL) gene at 11q23 mutated ($MLL^+$ leukaemia); these mutations occur in utero (Ford et al, 1993).

Spatial clustering in the UK was found to be concentrated in ALL in the youngest age group (0–4 years rather than 5–14 years,

Draper, 1991). Population mixing appears to be somewhat more strongly associated with CL for this age (Kinlen, 1995; Kinlen et al, 1990) but has seldom been examined separately for ALL. Few epidemiological studies and none of clustering or population mixing have considered common ALL separately.

The Hong Kong Paediatric Haematology and Oncology Study Group (HKPHOSG) has assembled a high quality data set with full population ascertainment and cell type for CL for the period 1984–90. Immunophenotype is available for the majority of ALL cases. The present analysis exploits these data to investigate spatial clustering of CL and relevant subgroups and the effects of population mixing on incidence and clustering. Hong Kong is particularly suitable for this study as it combines features (Shung, 1993) otherwise associated only with developed countries (affluence, high standards of hygiene and health care, low infant mortality) or only with developing countries (household crowding and high population density).

'Hong Kong' protectorate includes the original settlements of Hong Kong island and Kowloon (4347 hectares) and the New Territories (95 171 hectares, the Lands Department, Hong Kong Government, 1993). In 1973, the New Territories (NT) Development Department began a massive programme of building new towns to meet the needs of the expanding population of the protectorate and also to rectify the imbalance in population density between the NT and elsewhere. New Towns were formed by building both on existing rugged terrain and also land reclaimed from the sea. The first, Tsuen Wan, was already established in 1979. Elsewhere, the NT were then undeveloped, but the population has increased by over seven-fold from 1979 to 1989 and 315 000 housing units have been completed (Census and Statistics Department, Hong Kong, 1995). After Tsuen Wan, the largest New Town is Sha Tin, the population of which increased from 30 000 in 1973 to 500 000 in 1989 while, at the same time, a new transport infrastructure supported commuting to Kowloon and elsewhere in the New Territories (Hong Kong Yearbook, 1989). The initial intention was to house 1.8 million people in the New Towns but the programmes have been extended and 2.3 million were housed by the end of 1991.

In 1986, the Development Department extended its role to cover further development in urban areas, including parts of Hong Kong Island and Kowloon, where redevelopment of dilapidated urban areas and land reclamation provides substantial new opportunities. Altogether, between 1979 and 1989, 740 000 new housing units were constructed – more than one for every eight members of the population (Census and Statistics Department, Hong Kong, 1985).

It is clear that the extreme population mixing in Hong Kong offers an opportunity for further exploration of its impact on childhood leukaemia.

### METHODS

### Numerator data

These have been assembled by the HKPHOSG using in-patient records from all relevant hospitals in Hong Kong. The data were carefully checked by the individual paediatricians and again by computer programs run before analysis, with cases sorted by date of birth, date of diagnosis and place of diagnosis respectively. Three children whose surname indicated that they were Caucasian have been excluded as the registration of such children might not have been complete.

Families of children with leukaemia in Hong Kong are frequently rehoused following diagnosis, and the use of later addresses could have generated artifactual clustering. To avoid this, all addresses were carefully rechecked by paediatricians at each individual hospital to confirm that addresses analysed were those in which the children resided at the time of diagnosis.

### Immunophenotyping

Leukaemia samples were immunophenotyped by either immunofluorescence or immunocytochemistry using established methods (Chan et al, 1985). Laboratory records of immunophenotyping were available for 150 (73%) of the ALL cases. Each case was reviewed by one expert (LCC) and categorized into immunophenotypes according to the pattern of expression of surface markers (Chan et al, 1985). B-lineage markers used included CD22, CD19, CD10, CD20, CD21; T-lineage markers included CD2, CD3, CD4, CD5, CD7, CD8 and myeloid markers included CD13, CD14, CD15 and CD33. The following immunological subtypes were identified:

1 Early (Pro)B = CD10 negative and at least one additional B-lineage marker positive (>25% cells reactive with antibody).
2 Common ALL (cALL) = CD10 positive and at least one additional B-lineage marker positive.
3 B lineage = only one B-lineage marker positive (excluding CD10).
4 B-ALL = at least one B-lineage marker positive and $SIg^+$ and $\kappa$ or $\lambda$ positive.
5 ALL uncertain/unclassifiable = inadequate typing or only DR expressed.
6 T-ALL = at least two T-lineage markers positive.

There were two cases classified as common ALL expressing myeloid markers (i.e. $CD13^+$ cALL; $CD15^+$ cALL) and a case of biophenotypic leukaemia (i.e. $CD13^+CD33^+CD19^+$).

### Denominator data

These have been taken directly from the 1981 and 1991 censuses of Hong Kong and 1986 mid-census estimates provided by the Hong Kong Government Census and Statistics Department. Counts of total child–years at risk were computed from the 1981, 1986 and 1991 figures in two ways. Firstly, these figures were taken as accurate counts by sex and 5-year age group for 1979–83, 1984–88 and 1989–93 (i.e. for the 5-year period surrounding each census). The results reported here use this method but linear interpolation was also applied and similar results obtained.

### Geographical referencing

The smallest area unit for which census population denominators are available is the tertiary planning unit (TPU), and all case addresses were assigned manually to the TPU valid at their time of diagnosis. TPUs were not suitable as area units for analysis across the extended time period as changing of boundaries, splitting and aggregating had occurred, and some census counts were available only for combinations of TPUs. The present analysis is based on 120 'TPU groups' which have been formed by aggregating TPUs to form areas which are (a) stable from 1981 to 1991, (b) have population counts available for 1981, 1986 and 1991 and (c) are

 © Cancer Research Campaign 1997

single TPUs whenever possible. The process of aggregating TPUs was conducted *before* inspection of incidence data.

### Population growth

Population growth for 1981–86, 1981–91 and 1986–91 has been computed and TPU groups ranked using each of these values in turn. The ranked TPU groups were then divided into ten categories with approximately equal total child–years at risk in the period 1984–90. TPU groups in the the tenth decile were selected a priori as areas with extreme population increases. As excesses of childhood leukaemia are predicted *after* population mixing the present analyses have focused on areas with extreme population growth 1981–86 (six TPU groups in the highest decile with 13-fold to 65-fold population increases).

### Statistical methods

Age-standardized incidence rates have used the world standard population as reference (Parkin et al, 1988). Elsewhere (internal) indirect-standardization has been applied with expected numbers for small areas computed from overall age- and sex-specific incidence rates for Hong Kong for 1984–90. Some analyses consider non-standard age ranges. The approach has always been to apply age and sex standardization using reference rates for the portions of these ranges which lie within the standard 5-year groupings. To estimate population denominators it has been necessary to assume that the age distribution was uniform within each of the 5-year groups.

Standardized morbidity ratios (SMR=observed/expected × 100) quantify risk by area, and the Poisson distribution has been applied to test for significant departures of SMRs from 100.

The method of analysis of spatial clustering uses the Potthoff–Whittinghill method (Potthoff and Whittinghill, 1966*a*, *b*). This test for extra-Poisson variation between small areas is applicable when means (under the null hypothesis) vary on account of differences in the total population-at-risk and has performed well in a methodological study (Alexander and Boyle, 1996) when compared with other methods, including those which are 'boundary free' (Draper, 1991). The test conditions on the total number of cases observed ($O_+$) and takes as null hypothesis multinominal allocation of these cases to the ($n$) small areas with multinominal probabilities proportional to the expected numbers $\{E_j\}^n_{j=1}$. The test statistic (based on numbers of pairs of cases in the small areas) is

$$PW = \sum_{j=1}^{n} \frac{O_j(O_j - 1)}{E_j} \qquad (1)$$

The mean and variance of (1) under the null hypothesis are respectively

$$\mu = (O_+ - 1)$$
$$\sigma^2 = 2(n-1)\,\mu / O_+$$

The standardized form of the test statistic (reported here as *PW-Z*) is then

$$(PW - \mu)/\sigma$$

This is (asymptotically) a standardized normal deviate but all *P*-values here have been derived by Monte Carlo simulation with

**Figure 1** ALL incidence rates (Hong Kong, 1984–90)

**Figure 2** Proportions of common ALL by age (Hong Kong, 1984–90)

observed numbers of cases randomly allocated to the small areas with probabilities proportional to the expected numbers. The results show that use of simulation is particularly important when small numbers of cases and areas are available for analysis. A similar test (Potthoff and Whittinghill, 1966*a*) has been used to check for spatial homogeneity of the probability that haematology reports of immunophenotyping tests were available for ALL cases.

All analyses have been conducted for total childhood leukaemia, ALL and common ALL for the entire age range (0–14 years) and for the conventional representation of the childhood peak (0–4 years). In addition, we wished to use age groups which were biologically more appropriate. The range selected a priori was from 18 months up to the seventh birthday with the lower bound chosen to exclude so far as possible MLL$^{+ve}$ leukaemias (Greaves, 1996). Alternative age bands for the childhood peak were included subsequently (a) because most cancer registries provide childhood leukaemia incidence data only in completed years and (b) to check for sensitivity of our results to the particular

© Cancer Research Campaign 1997 

**Table 1** Childhood leukaemia incidence in Hong Kong, 1984–90

| | Age range | | | |
|---|---|---|---|---|
| | 0–4 years | 5–9 years | 10–14 years | All[a] |
| ALL | | | | |
| Observed number | 99 | 75 | 31 | 205 |
| Rate $10^{-5}$ | 3.78 | 2.61 | 1.04 | 2.53[a] |
| Total leukaemia | | | | |
| Observed number | 121 | 98 | 42 | 261 |
| Rate $10^{-5}$ | 4.61 | 3.42 | 1.14 | 3.21[a] |

[a]Rates here are directly standardized to the world standard population.

**Table 2** Spatial clustering of childhood leukaemia within TPU-groups, 1984–90: *PW-Z*[a] (*P*[b])

| | Diagnostic group | | |
|---|---|---|---|
| Age | ALL | Common ALL | Total leukaemia |
| 0–14 years | –0.33 | –1.13 | –0.88 |
| | (–) | (–) | (–) |
| 0–4 years | 1.34 | 0.17 | 1.05 |
| | (0.09) | (–) | (0.14) |
| Childhood peak (2–6 years) | 2.14 | 1.10 | 2.03 |
| | (0.028) | (0.14) | (0.032) |

[a]Potthoff–Whittinghill *Z*; under the null hypothesis this is (asymptotically) a standardized normal deviate. [b]Devised by Monte Carlo simulation (99 999 runs) with observed numbers of cases allocated at random to appropriate age–sex subgroups of the population-at-risk.

choices of age. The choices, influenced by the observed age–incidence curve and frequency of common ALL (reported in Figures 1, 2) were 2–4 years and 2–6 years, both inclusive. The results with 18 months and 2 years respectively as lower end were almost identical. Therefore, for simplicity and to facilitate comparison with other data, the results recorded here for the childhood peak are for diagnoses at ages 2–6 years.

Our predictions were that clustering and associations with population mixing would be concentrated in ALL, the 0–4 years group, the childhood peak and the common sub-type. Certain other groups (acute myeloid leukaemia, older onset disease and T-cell ALL) were analysed for comparison purposes but results are not reported in detail.

Fortran programs and the SPSS statistical package were used in these analyses.

### RESULTS

Incidence rates of total childhood leukaemia and ALL (Table 1) show the marked childhood peak of ALL (Figure 1) but it is both broader and somewhat older than is usual in developed Caucasian populations. The usual male predominance was also seen with an overall male–female ratio of 1.4:1.0. Cell type was available for all cases with the majority (78%) being ALL; immunophenotype was known for 73% of ALL cases and, of these, 59% were common ALL. The relative frequency of common ALL was low under the age of 18 months (Figure 2). The test of spatial variation of availability of immunophenotypic classification showed no evidence of heterogeneity (chi square = 31.9 on 40 d.f., $P > 0.5$).

The Potthoff–Whittinghill test found evidence of overall spatial clustering (Table 2) for ALL in the youngest age group and in the childhood peak, for which conventional levels of statistical significance were attained. Similar, though weaker, results were obtained for total leukaemia. The distributions of cases at older ages were more uniform than predicted by Poisson variability and, for the entire age range, there was no evidence of clustering. The analyses for common ALL show a similar homogeneity for the entire age range and heterogeneity for the childhood peak, but the latter is weaker. This is likely to be attributable to the small numbers available for analyses and the incomplete availability of immunophenotypic classification.

In areas in the highest deciles of population growth (Table 3), incidence for the age/diagnosis groups of interest was elevated but

**Table 3** Childhood leukaemia 1984–90 in small areas of Hong Kong which have experienced extreme population growth[a]

| | | Incidence | | | | Clustering | |
|---|---|---|---|---|---|---|---|
| Diagnostic group | Age range[b] | Obs | Exp[c] | SMR | *P*[d] | PW-Z[e] | *P*[f] |
| ALL | 0–14 years | 25 | 23.07 | 108 | – | 1.50 | 0.08 |
| | 0–4 years | 14 | 11.10 | 126 | 0.23 | 3.66 | 0.006 |
| | 2–6 years | 15 | 10.68 | 140 | 0.12 | 3.50 | 0.007 |
| Common ALL | 0–14 years | 10 | 9.70 | 103 | – | 1.74 | 0.06 |
| | 0–4 years | 7 | 4.71 | 149 | 0.20 | 1.20 | 0.12 |
| | 2–6 years | 9 | 4.97 | 181 | 0.07 | 2.42 | 0.029 |
| Total leukaemia | 0–14 years | 30 | 29.34 | 102 | – | 1.37 | 0.09 |
| | 0–4 years | 15 | 13.56 | 111 | 0.39 | 3.00 | 0.013 |
| | 2–6 years | 16 | 12.23 | 131 | 0.17 | 2.98 | 0.014 |

[a]The top ten per cent (of person–years at risk) when TPU groups are ranked by percentage growth of childhood population 1981–86. [b]All age ranges are inclusive. [c]Expected numbers derived by applying overall Hong Kong age- and sex-specific incidence rates to the local population. [d]Poisson *P*-values (two-sided).
[e]Potthoff–Whittinghill *Z*; under the null hypothesis this is (asymptotically) a standardized normal. [f]*P*-values derived by Monte Carlo simulation (99 999 runs) with observed numbers of cases allocated at random to appropriate age–sex subgroups of the population at risk.

© Cancer Research Campaign 1997

**Table 4** Spatial clustering of childhood leukaemia in the New Territories[a] of Hong Kong 1984–90: *PW-Z*[b] (*P*)[c]

| Age[d] | Diagnostic group ALL | Common ALL | Total leukaemia |
|---|---|---|---|
| 0–14 years | −0.11 | 0.42 | −0.12 |
| | (–) | (–) | (–) |
| 0–4 years | 2.36 | 0.40 | 2.55 |
| | (0.022) | (–) | (0.017) |
| 2–6 years | 2.37 | 1.58 | 2.56 |
| | (0.022) | (0.07) | (0.016) |

[a]Excluding Tsuen Wan which was already established in 1979. [b]Potthoff–Whitinghill *Z*; under the null hypothesis this is (asymptotically) standardized normal. [c]Devised by Monte Carlo simulation (99 999 runs) with observed numbers of cases allocated at random to appropriate age–sex subgroups of the population-at-risk. [d]All age ranges are inclusive.

**Table 5** Childhood leukaemia at ages 2–4 years, Hong Kong, 1984–90

| Area | Diagnostic group | Incidence Obs | Exp[a] | SMR | *P*[b] | Clustering PW-Z[c] | *P*[d] |
|---|---|---|---|---|---|---|---|
| Total | ALL | 67 | 67 | – | – | 3.09 | 0.007 |
| | Common ALL | 31 | 31 | – | – | 1.45 | 0.08 |
| | Total leukaemia | 75 | 75 | – | – | 3.43 | 0.004 |
| Extreme growth[e] | ALL | 11 | 7.51 | 146 | 0.14 | 3.71 | 0.005 |
| | Common ALL | 7 | 3.48 | 201 | 0.06 | 1.20 | 0.12 |
| | Total leukaemia | 11 | 8.41 | 131 | 0.23 | 3.71 | 0.006 |
| NT[f] | ALL | 23 | 22.06 | 104 | 0.45 | 2.90 | 0.012 |
| | Common ALL | 13 | 10.20 | 128 | 0.23 | 0.17 | 0.20 |
| | Total leukaemia | 26 | 24.70 | 105 | 0.42 | 3.95 | 0.003 |

[a]Expected numbers derived by applying overall Hong Kong age- and sex-specific rates to the local population. [b]Poisson *P*-values (two-sided). [c]Potthoff–Whittinghill *Z*; under the null hypothesis this is asymptotically standardized normal. [d]*P*-values derived by Monte Carlo simulation (99 999 runs) with observed numbers of cases allocated at random to appropriate age–sex subgroups of the population at risk. [e]The top ten per cent (of person–years at risk) when TPU groups are ranked by percentage growth of childhood population 1981–86. [f]Excludes Tsuen Wan.

did not differ significantly from that in other areas. There was strong evidence of spatial clustering between these TPU groups, and this is independent of the overall excess incidence. When the same analyses were applied to the comparison groups, the standardized Potthoff–Whittinghill score never exceeded 0.9 (data not shown). Application of the Potthoff–Whittinghill test to the rest of Hong Kong revealed no significant evidence of clustering.

The results for spatial clustering, and especially those in the areas of extreme population growth, are dominated by excess incidence in one TPU group, which was the only one in the highest decile for population growth in each of the three time periods: 1981–86 (three-fold increase), 1986–91 (2.5-fold increase) and 1981–91 (13-fold increase). Altogether ten cases of CL lived in this small area and of these nine were ALL (five of which were common ALL with ages from 31 to 60 months). This TPU group is in the New Territories (NT) and mainly comprised new housing estates built in rural areas.

Application of the statistical analyses to the 32 TPU groups in the NT (outside Tsuen Wan) found significant spatial clustering for each of the groups of primary interest (Table 4). Most SMRs were around 100 (95–105); those for common ALL in the childhood peak were somewhat elevated (117–118) but did not differ significantly from 100. Application of the Potthoff–Whittinghill test to the rest of Hong Kong found very little evidence of clustering.

Alternative age bands were considered for the childhood peak (see Methods). The low percentage of common ALL in children under 18 months (Figure 2) indicates that the lower age should not be reduced to 1 year. The age-specific incidence (Figure 1) suggests that the fifth birthday may be a more appropriate upper bound. Results of all tests of clustering when applied to the age range 18 months–6 years (data not shown) are very similar to those for 2–6 years. Analyses of the more restricted group (2–4 years) showed increased evidence of clustering (Table 5). Thus, our results are not dependent on a precise definition of the childhood peak in terms of age at diagnosis.

### DISCUSSION

Although the age-standardized incidence rates reported here are around 10% lower than those commonly found in Western Europe and North America, the overall pattern is quite typical of CL in developed countries (Linet, 1991). In particular, the usual ALL – total acute leukaemia ratio, the marked childhood peak of ALL, the male predominance and the high frequency of common ALL are all present.

Particular interest attached to Hong Kong as a novel area in which to examine spatial clustering of CL and its association with population mixing as the large scale movements there have exposed many people to increases in population density. The demographic factors there, especially the high population density (5305 per km$^2$), differ from those in the UK where the majority of investigations of these issues have been conducted. Although infant mortality in Hong Kong is lower than in the UK (Shung, 1993), first exposure to some common infections occurs, on average, earlier in Hong Kong than in the UK (Kangro et al, 1994), as would be anticipated from the high population density and household crowding. The spectrum of paediatric infectious illness shows both similarity and differences (Davies, 1992). Urinary tract infections, acute respiratory tract infections and the rising incidence of asthma are similar to the UK but meningococcal meningitis is virtually absent despite the high population density.

We were, as usual, constrained to the use of small areas corresponding to census geography and these have variable size but, on average, contain around 42 000 people. The Potthoff–Whittinghill test is appropriate in these circumstances and has shown spatial clustering for the youngest age group of ALL, which replicates the UK experience (Alexander, 1991; Draper, 1991). Further examination associated clustering with the childhood peak of ALL and common ALL. The results are not very sensitive to the particular choice of age band for the childhood peak. The 2–4 years group did not represent a prior hypothesis, but we note that clustering of these cases was particularly strong.

We have no direct indicator of population mixing but it is clear that substantial movements of large populations do occur in Hong Kong when new housing estates are built, especially in the New Territories where population density was relatively low. Schools and kindergartens are normally specific to single housing estates. Many new housing estates are built on green-field sites, and our use of population increase as a proxy should identify these and correspond to the 'rural' New Towns of Kinlen et al (1990). We have found evidence of overall excess incidence in the CL

subgroups of interest in these 'extreme' areas but this is moderate, except for common ALL, and not statistically significant. These results confirm the observations of Kinlen and colleagues but extend them to a new demographic setting and refine them by their focus on ALL and biologically meaningful subgroups of ALL specified a priori.

Our results provide strong evidence of clustering concentrated in the areas where population mixing is likely, even after allowing for overall excess of incidence. The results apply to ALL in young children and have not been observed for subgroups of CL which exclude ALL in the childhood peak. As immunophenotype was not available for all ALL cases, we cannot exclude the possibility of geographical bias for the reported distribution of common ALL, but the evidence of spatial clustering in the extreme areas and its absence for, for example, T-cell ALL provides support for hypotheses linking common ALL with exposures to microepidemics of common infection(s). The concentration of common ALL in the one 'cluster' we have identified is consistent with this.

This is the first study to investigate formally clustering of CL within situations of population mixing, although a study of New Towns in the UK found excess incidence in each of those that were 'rural' (Kinlen et al, 1990). Systematic application of the Potthoff–Whittinghill test to the UK situations studied by Kinlen and colleagues would be very interesting and might distinguish *uniform and moderate* from *sporadic and extreme* elevation of risk. As the results of Kinlen et al have been interpreted in terms of population microepidemics of an infectious agent, the latter would be expected even if the agent were common. However, the pattern of infection in the population subsequent to mixing (and hence the pattern of leukaemia incidence) can be expected to depend critically on the prevalence of the infection and its geographical distribution as well as its geographical variability. If, for example, proportions of either susceptibles or infectives in Hong Kong were much lower than in the UK, then a pattern of spatial clustering unaccompanied by high overall rates would be predicted. This pattern describes our results at least for the New Territories.

Other explanations for the patterns we have observed are possible. These include chance, errors in population denominators (arising both in census counts and in subtleties of the age-specific population distribution) and the presence of other environmental leukaemogenic factors. It is difficult to see how any of these could have led to the concentration of the effects in precisely those CL subgroups for which a causative role for patterns of exposure to infection had been predicted a priori.

There is a substantial body of evidence suggesting an infective basis for the childhood peak of ALL, although it is not known whether this is likely to involve direct transformation or indirect effects of one or more viruses or even bacteria (Greaves and Alexander, 1993). The postulated abnormal immunological response appears to be HLA associated (Taylor et al, 1995) which may lead to the definition of candidate agents. Alternatively the application of DNA substraction techniques, similar to those which have identified HHV8 in cases of Kaposi's sarcoma (Moore et al, 1995), may help to resolve this key issue. Meanwhile, in the absence of candidate agents, epidemiologists must proceed indirectly. For example, case–control analyses of potentially infectious contacts in infancy (Petridou et al, 1993) and comparisons of international patterns of childhood leukaemia with ages of exposure to specific infections may help to refine the hypothesis. The present results emphasize the importance of conducting separate analyses (but with numbers sufficient to provide adequate statistical power) for the childhood peak of ALL and/or common ALL.

### ACKNOWLEDGEMENTS

The Leukaemia Research Fund supported FE Alexander during part of this study and her research assistant, Miss Kathleen Lee, during August–September 1994; this support is gratefully acknowledged. MF Greaves is supported by the Leukaemia Research Fund and the Kay Kendall Leukaemia Fund. The study was facilitated by British Council Academic Link exchanges between Hong Kong and the UK (LC Chan, FE Alexander and MF Greaves). Miss Lee is thanked for her computing assistance and Mrs Morag Leitch for typing the manuscript. Miss SF Chung is thanked for compiling the Hong Kong population data and TPU coding. Leo Kinlen is thanked for his perceptive comments on reading a preliminary draft of this manuscript.

### REFERENCES

Alexander FE (1991) Investigations of localised spatial clustering, and extra-Poisson variation. In *The Geographical Epidemiology of Childhood Leukaemia and Non-Hodgkin Lymphomas in Great Britain, 1966–83* Draper G. (ed) pp. 69–73. HMSO: London

Alexander FE and Boyle P (eds) (1996) *Statistical Methods of Investigating Localised Clustering of Disease*. IARC: Lyon (in press)

Census and Statistics Department (1985). *Hong Kong Monthly Digest of Statistics*. Census and Statistics Department: Hong Kong.

Chan LC, Pegram SM and Greaves MF (1985) Contribution of immunophenotype to the classification and differential diagnosis of acute leukaemia. *Lancet* **2**: 475–479

Davies DP (1992) Paediatric illness in Hong Kong and Britain. *Arch Dis Child* **67**: 543–549

Doll R (1989) The epidemiology of childhood leukaemia. *J Royal Stat Soc A* **152**: 341–351

Draper G (ed.) (1991) *The Geographical Epidemiology of Childhood Leukaemia and Non-Hodgkin's Lymphomas in Great Britain*, 1966–83. OPCS: London

Ford AM, Ridge A, Cabrera ME, Mahmoud H, Steel CM, Chan LC and Greaves M. (1993) In utero rearrangements in the trithorax-related oncogene in infant leukaemias. *Nature* **363**: 358–360

Gardner MU, Snee MP, Hall AJ, Powell CA, Downes S and Terrell JD (1990) Results of a case–control study of leukaemia and lymphoma among young people near Sellafield nuclear plant in West Cumbria. *Br Med J*, **300**: 423–429

Greaves MF (1996) Workshop report. Infant leukaemia biology, aetiology and treatment. *Leukaemia* **10**: 372–377

Greaves MF and Alexander FE (1993) An infectious etiology for common acute lymphoblastic leukemia in Childhood? *Leukemia* **7**: 349–360

Greaves MF, Pegram SM and Chan LC (1985) Collaborative group study of the epidemiology of acute lymphoblastic leukaemia subtypes: background and first report. *Leuk Res* **9**: 715–733

Greaves MF, Colman SM, Beard MEJ, Bradstock K, Cabrera ME, Chen P-M, Jacobs P, Lam-Po-Tang PRL, Macdougall LG, Williams CKO and Alexander FE. (1993) Geographical distribution of acute lymphoblastic leukaemia subtypes: second report of the Collaborative Group Study. *Leukemia* **7**: 27–34

Heath CW and Hasterlick RJ (1963) Leukaemia amongst children in a suburban community. *Am J Med* **34**: 796–812

Hong Kong Government (1989) Hong Kong Yearbook

Kangro HO, Osman HK, Lau YL, Heath RB, Yeung CY and Ng MH (1994) Seroprevalence of antibodies to human herpes viruses in England and Hong Kong. *J Med Virol* **43**: 91–96

Kinlen LJ (1993) Childhood leukaemia and non-Hodgkins lymphoma in young people living close to nuclear reprocessing sites. *Biomed Pharmacother* **47**: 429–434

Kinlen LJ (1995) Epidemiological evidence for an infective basis in childhood leukaemia *Br J Cancer* **71**: 1–52

Kinlen LJ, Clarke K and Hudson C (1990) Evidence from population mixing in British New Towns 1946–85 of an infective basis for childhood leukaemia. *Lancet* **336**: 557–582

Kinlen LJ, Hudson C and Stiller LA (1991) Contacts between adults as evidence for an infective origin of childhood leukaemia: an explanation for the excess near nuclear establishments in Wester Berkshire. *Br J Cancer* **64**: 549–554

© Cancer Research Campaign 1997

Kinlen LJ, O'Brien F, Clarke K, Balkwill A and Matthews F (1993) Rural population mixing and childhood leukaemia: effects of the North Sea oil industry in Scotland, including the area near Dounreay nuclear site. *Br Med J* **306**: 743–748

Knox EG (1994) Leukaemia clusters in childhood: geographical analysis in Britain. *J Epidemiol Comm Health* **48**: 369–376

Lagakos SW, Wessen BJ and Zelen M (1986) An analysis of contaminated well water and health effects in Woburn, Massachusetts. *J Am Stat Assoc* **81**: 583–596

Linet MS and Devesa SS (1991) Descriptive epidemiology of childhood leukaemia. *Br J Cancer* **63**: 424–429

MacMahon B (1992) Leukaemia clusters around nuclear facilities in Britain. *Cancer Causes Controls* **3**: 283–288

Moore PS and Chang Y (1995) Detection of Herpesvirus-like DNA sequences in Kaposi's sarcoma in patients with and those without HIV infection. *New Engl J Med* **332**: 1181–1185

Mulder YM, Frijver M and Kreis IA (1994) Case–control study on the association between a cluster of childhood haematopoietic malignancies and local environmental factors in Aalsmeer, The Netherlands. *J Epidemiol Comm Health* **48**: 161–165

Parkin DM, Stiller CA, Draper GJ, Bieber CA, Terracini B and Young JL (eds) (1988) International Incidence of Childhood Cancer. World Health Organization. IARC Scientific Publications No. 87: Lyon

Petridou E, Kassimos E, Kalmanti M, Kosmidis H, Haidas S, Flytzani V, Tong D and Trichoppulous D. (1993) Age of exposure to infections and risk of childhood leukaemia. *Br Med J* 307: 774

Potthoff RF and Whittinghill M (1966*a*) Testing for homogeneity. I. The binomial and multinominal distributions. *Biometrika* **53**: 167–182

Potthoff RF and Whittinghill M (1966*b*) Testing for homogeneity. II. The Poisson distribution. *Biometrika* **53**: 183–190

Ramot B and Magrath I (1982) Hypothesis: the environment is a major determinant of the immunological sub-type of lymphoma and acute lymphoblastic leukaemia in children. *Br J Haematol* **52**: 183–189

Ross JA, Potter JD and Robinson LL (1994) Infant leukaemia, topoisomerase II inhibitors, and the MLL gene *J Natl Cancer Inst* **86**: 1678–1680

Shung E (1993) *Decline in Infant Mortality in Hong Kong 1961–1991*. Public Health and Epidemiology Bulletin. Department of Health: Hong Kong

Taylor GM, Robinson MD, Binchy A, Birch JM, Stevens RF, Jones PM, Carr T, Deardon S and Gokhale DA. (1995) Preliminary evidence of an association between HLA-DPB1 *0201 and childhood common acute lymphoblastic leukaemia supports an infectious aetiology. *Leukemia* **9**: 440–443

© Cancer Research Campaign 1997